# Correlation of preoperative sonographic staging and postoperative histopathologic staging in patients with invasive breast cancer

**DOI:** 10.1007/s00404-024-07699-5

**Published:** 2024-09-02

**Authors:** Carolin Mueller, Julia Sarah Maria Zimmermann, Marc Philipp Radosa, Anna Katharina Hahn, Askin Canguel Kaya, Sarah Huwer, Lisa Stotz, Gudrun Wagenpfeil, Christoph Georg Radosa, Erich-Franz Solomayer, Julia Caroline Radosa

**Affiliations:** 1 Department of Gynecology, Obstetrics, and Reproductive Medicine, Saarland University Medical Center, 66421 Homburg, Saar, Germany; 2 Outcomes Research Consortium, Department of Anesthesiology, Cleveland Clinic, Cleveland, OH 44195 USA; 3 Department of Gynecology and Obstetrics, Klinikum Bremen-Nord, Bremen, Germany; 4 Department of Obstetrics & Gynecology, Medical Center–University of Freiburg, 79106 Freiburg, Germany; 5 Institute for Medical Biometry, Epidemiology and Medical Informatics (IMBEI), Saarland University, Campus Homburg, 66421 Homburg, Germany; 6 Institute and Polyclinic of Diagnostic and Interventional Radiology, Medical University, TU Dresden, Dresden, Germany

**Keywords:** Breast cancer, Sonography, Accuracy, HER2 positive, Breast imaging, Breast cancer diagnostics

## Abstract

**Purpose:**

To assess the accuracy of preoperative sonographic staging in patients with primary invasive breast cancer.

**Methods:**

We retrospectively analyzed a prospectively kept service database of patients with newly diagnosed, unifocal, cT1-3, invasive breast cancer. All patients were diagnosed at a single center institution between January 2013 and December 2021. Clinical T stage was assessed preoperatively by ultrasound and correlated with the definite postoperative pathologic T stage. Demographics, clinical and pathological characteristics were collected. Factors influencing accuracy, over- and underdiagnosis of sonographic staging were analyzed with multivariable regression analysis.

**Results:**

A total of 2478 patients were included in the analysis. Median patients’ age was 65 years. 1577 patients (63.6%) had clinical T1 stage, 864 (34.9%) T2 and 37 (1.5%) T3 stage. The overall accuracy of sonography and histology was 76.5% (*n* = 1896), overestimation was observed in 9.1% (*n* = 225) of all cases, while underestimation occurred in 14.4% (*n* = 357) of all cases. Accuracy increased when clinical tumor stage cT was higher (OR 1.23; 95% CI 1.10–1.38, *p* ≤ 0.001). The highest accuracy was seen for patients with T2 stage (82.8%). The accuracy was lower in Luminal B tumors compared to Luminal A tumors (OR 0.71; 95% CI 0.59–0.87, *p* ≤ 0.001). We could not find any association between sonographic accuracy in HER2 positive patients, and demographic characteristics, or tumor-related factors.

**Conclusion:**

Our unicentric study showed a high accuracy of sonography in predicting T stage, especially for tumors with clinical T2 stage. Tumor stage and biological tumor factors do affect the accuracy of sonographic staging.

## What does this study add to the clinical work

**Table:** 

Our unicentric study showed a high accuracy of sonography in predicting T stage, especially for tumors with clinical T2 stage (82.8%). In the subgroup of HER2 positive patients, overall accuracy of sonography and histology was 79.2%.	

## Introduction

Breast cancer stands as the most prevalent malignancy worldwide. In 2020, the World Health Organization (WHO) reported that 2.3 million people were diagnosed with breast cancer globally [1]. It is assumed that the incidence will continue to rise and that around 3 million people will be diagnosed per year by 2040 [1]. For this reason, physicians and scientists are continually working to optimize prophylaxis, diagnostics, and therapy strategies.

Crucial factors guiding therapy decisions include tumor size, grading, the proliferation marker Ki67, patient age, genetic risk factors, results of gene expression tests, and any prior medical conditions that could impact treatment choices [2]. However, the clinical tumor size (T) of invasive breast carcinoma, alongside regional lymph node involvement (N) considered within the TNM staging system, remains one of the most critical factors influencing prognosis and subsequent therapy decisions [3]. For this reason, accurate determination of tumor size and lymph node involvement preoperatively is of high importance. Moreover, the tumor subtype plays a crucial role. Especially patients with HER2 positive or triple negative tumors are candidates for neoadjuvant chemotherapy dependent on clinical tumor size [4].

Radiologic tumor staging prior to surgery may consist of ultrasound, mammography, magnetic resonance imaging, and computed tomography [4]. The gold standard imaging techniques for preoperative assessment are mammography and ultrasound, as they provide a high correlation with postoperatively pathological tumor size [5]. However, biological tumor features and clinical tumor size might affect the accuracy [5]. Since ultrasound is readily available, cost-effective, and minimally invasive, it is commonly employed as the initial diagnostic method for distinguishing between malignant and benign masses [6]. Moreover, the utilization of color Doppler can be beneficial in the diagnostic process [6].

However, all imaging methods frequently struggle to accurately differentiate between the extent of invasive carcinoma and the presence of associated in situ carcinoma or related inflammatory processes [7, 8]. Consequently, they may either overestimate or underestimate the clinical tumor stage [7]. As breast-conserving surgical techniques advance and neoadjuvant systemic therapies become more common in treating breast cancer, the clinically assessed tumor size plays a crucial role. It not only influences the choice of surgical procedure for patients but also aids in determining whether neoadjuvant therapy is appropriate [2].

Incorrect preoperative estimation of breast lesion size can therefore lead to unnecessary neoadjuvant chemotherapy, incomplete resection or necessitate tumor re-excision. Regarding determination of clinical overestimation or underestimation of tumor stage, earlier research concentrated on absolute tumor size, regarding a variance of 2 mm [9] to 10 mm [10] to 20 mm [11]. However, what holds greater significance than the physical dimensions of the lesions are the clinical T stages, which account for microinvasion and the extent of spread to the chest wall or skin [3]. Moreover, the clinical T stage is the basis for subsequent treatment decisions [2, 4, 12].

Hence, our aim was to assess the accuracy of sonographic T-staging and identify factors linked to the agreement between sonographic and pathological T-stage in patients diagnosed with primary breast cancer.

## Materials and methods

This study was conducted as a retrospective analysis of a prospectively maintained service database of women presenting with newly diagnosed invasive breast cancer. Data were collected from January 2013 to December 2021 at the department of Gynecology, Obstetrics and Reproductive Medicine of Saarland University Medical Center. The study was approved by the ethical committee of the Medical Association of Saarland (study # 33/24, date of approval 3/6/2024). Parts of the present registry were previously analyzed to assess the effectiveness of preoperative sonographic staging in predicting limited axillary disease (one or two metastatic lymph nodes) [13].

In the present study, patients with primary, unifocal, unilateral breast cancer, clinical tumor stage cT1-3, age ≥ 18 years who had received preoperative sonographic tumor staging (sonography and percutaneous biopsy) and who underwent surgery for breast cancer at the Saarland University Medical Center with complete data in their charts concerning the target variables were included. Patients with distant metastases (M1), solely carcinoma in situ disease, multicentric or bilateral carcinomas and patients who received neoadjuvant chemotherapy were excluded. As tumor stage cT4 is typically diagnosed based on clinical indicators (e.g., cutis or muscle infiltration) rather than solely on sonographic tumor size measurement, cT4 tumors were omitted from the analysis.

Demographics (e.g., age and BMI), as well as clinical and pathologic data were collected. Recorded data included pre- and postoperative tumor stage following the TNM classification of malignant tumors, eighth edition [14] including tumor (T) and nodal status (N), grading (Elston and Ellis 1991 [15]), and immunohistochemistry for determination of estrogen receptor (ER), progesterone receptor (PR), HER2 and Ki67 [16, 17]. Subtypes were defined by immunohistochemistry in accordance with St. Gallen classification [18]: Luminal A (ER ± , PR ± , HER2 −, Ki67 ≤ 15%), Luminal B (ER ± , PR ± , HER2 ± , Ki67 > 15%), HER2 positive (ER −, PR −, HER2 +) and triple negative (ER −, PR −, HER2 −). HER2 + receptor status was determined if they scored 3 + by immunohistochemistry (IHC) or in case of a HER2 score of 2 + , and positive fluorescence in situ hybridization (FISH).

### Sonography

Bilateral whole-breast ultrasound was performed by board-certified breast physicians in sagittal and transverse planes with knowledge of clinical findings. All examinations were conducted by a hand-held technique using a Voluson E8/10 (GE Healthcare, Chicago, IL, USA) and Hitachi Hi Vision Ascendus (Hitachi, Tokyo, Japan) devices equipped with 5–12 MHz and 13–3 MHz linear-array transducers. The measurements of tumor’s dimension were obtained according to the American College of Radiology [19] and incorporated the echo-poor center of the lesion and the echogenic halo and were obtained in sagittal, transverse, and anteroposterior planes. The largest diameter in any plane was defined as the sonographic tumor size (in mm) according to our department protocol. The pathologic tumor size was defined as the largest diameter (in mm) of formalin-fixed pathologic tumor samples. Accuracy of sonography in establishing tumor size was evaluated by comparing preoperative images with postoperative pathologic findings regarding tumor stage and was graded as accordance, underestimation (defined as sonographic T stage lower than pathologic T stage) and overestimation (defined as sonographic T stage higher than pathologic T stage). Accuracy of sonography was correlated with tumor stage, tumor histology, tumor subgroup, and patients’ characteristics (age, BMI).

### Statistical analysis

For the statistical analyses, SPSS 29.0 (IBM, Armonk, USA) was used. The Kolmogorov–Smirnov test was used to test for normal distribution in quantitative parameters. Consequently, quantitative parameters are presented as mean with standard deviation (if normally distributed) or median with minimum and maximum (if not normally distributed). Qualitative parameters are presented as absolute frequencies and percentages. Multiple logistic regression was performed to associate possible influencing factors (e.g., tumor stage, tumor histology, tumor subgroup, and patients’ characteristics) with accuracy of sonographic and pathologic tumor stage. Binary logistic regression was first performed univariable. All variables that provided statistically significant results were afterwards included in a multiple logistic regression. The analysis was initially conducted for all patients and subsequently repeated for HER2-positive patients only. As treatment recommendations vary for HER2 positive patients, particularly based on preoperative tumor size (neoadjuvant chemotherapy vs. surgery first).

## Results

**Fig. 1:**
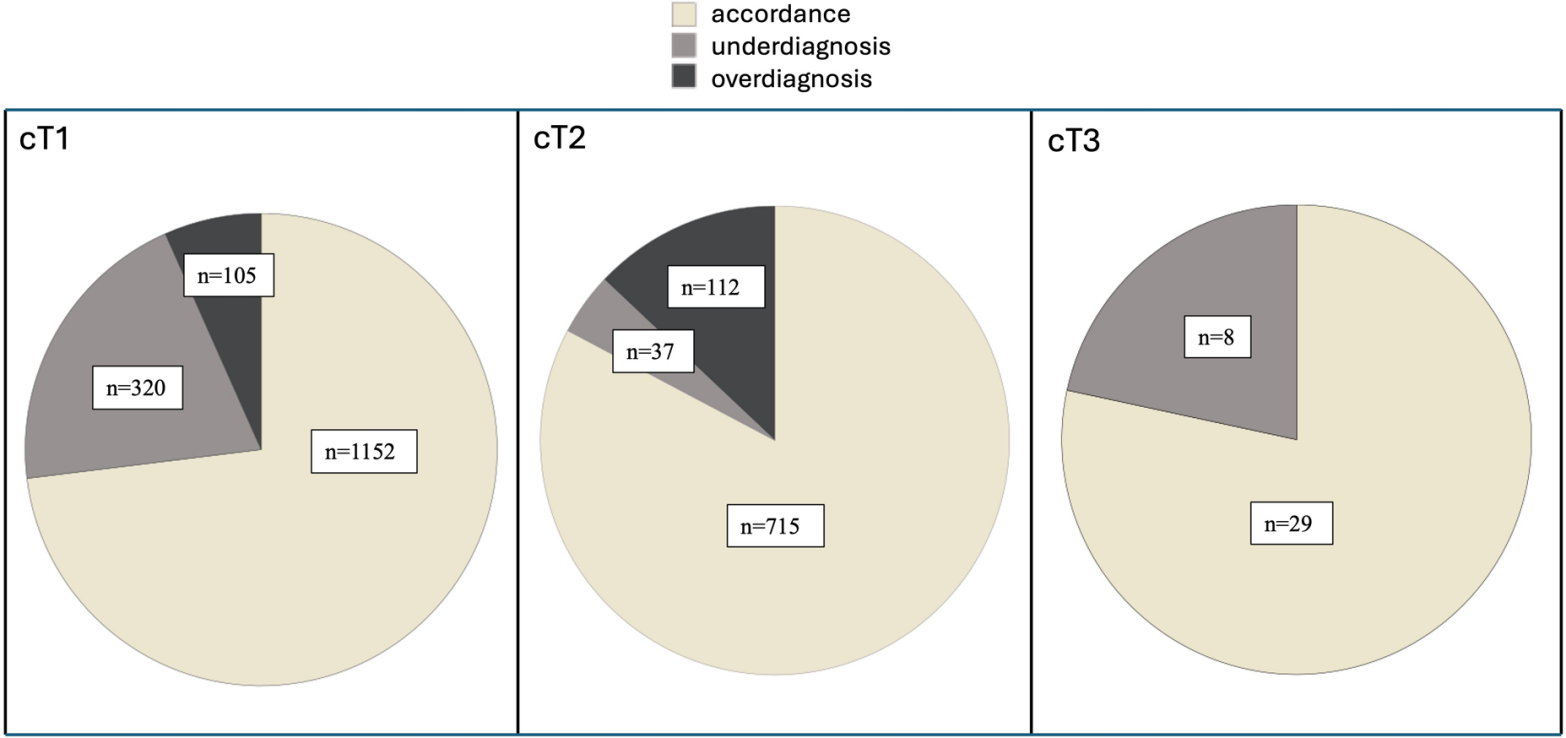
Accordance, underdiagnosis and overdiagnosis in cT1, 2 and 3 categories

A total of 2478 patients were included in the analysis. Median patients’ age was 65 years (min. 26, max. 95), and median BMI was 25.6 kg/m^2^ (min. 13, max. 49). Clinical and pathological tumor stages, as well as their accordance and over-/underestimation is reported in Table 1 and Fig. 1. 1577 patients (63.6%) had clinical T1 tumors, 864 (34.9%) T2 tumors, and 37 (1.5%) T3 tumors, respectively. The overall accuracy of sonography and histology was 76.5% (*n* = 1896), overestimation was observed in 9.1% (*n* = 225) of all cases, while underestimation occurred in 14.4% (*n* = 357) of all cases.

**Table 1:** Clinical and pathological tumor stages

Tumor stage	Clinical stage “cT” (sonography)	Pathological stage “pT” (histology)	Accordance	Underdiagnosis	Overdiagnosis
1a	72 (2.9%)	89 (3.6%)	55 (76.4%)	17 (23.6%)	0
1b	359 (14.5%)	368 (14.9%)	274 (76.3%)	64 (17.8%)	21 (5.8%)
1c	1146 (46.2%)	979 (39.5%)	823 (71.8%)	239 (20.9%)	84 (7.3%)
2	864 (34.9%)	972 (39.2%)	715 (82.8%)	37 (4.3%)	112 (13%)
3	37 (1.5%)	68 (2.7%)	29 (78.4%)	0	8 (21.6%)
4	0	2 (0.1)			
total	2478 (100%)	2478 (100%)			

Numbers are presented as frequencies (= *n*) and percentages (%). Accordance of tumor stage, underdiagnosis and overdiagnosis refers to clinical stage “cT”

Tumor subtype, grading, tumor histology and clinical stage cN are reported in Table 2. Most patients had Luminal A (*n* = 1106, 44.6%) or Luminal B (*n* = 1115, 45.0%) subtype, whereas HER2 positive tumors represented 3.1% (*n* = 77) and triple negative tumors 7.3% (*n* = 180) of cases. The predominant grading observed was G2 (*n* = 1666, 67.2%), and the prevailing subtype identified were NST (no special type) carcinoma (*n* = 1870, 75.5%). The majority of patients had no lymph node involvement (cN0, *n* = 1936, 78.1%). Multiple logistic regression was performed to analyze factors associated with accordance of sonographic and pathologic tumor stage (see Table 3). Accordance increased when clinical tumor stage cT was higher (OR 1.23, 95% CI 1.10–1.38; *p *≤ 0.001). Moreover, the accordance was lower in Luminal B tumors compared to Luminal A tumors (OR 0.71; 95% CI 0.59–0.87, *p* ≤ 0.001).

**Table 2:** Occurrence of tumor subtypes, grading, and clinical tumor stage cN presented with frequencies and percentages

Tumor subtype	Frequency (*n*) Total *n* = 2478	Percentage (%) 100%
Luminal A (ER ± PR ± , Her2 −, Ki67 < 15)	1106	44.6
Luminal B (ER ± PR ± , Her2 −, Ki67 ≥ 15)	1115	45.0
HER2 positive (ER ± PR ± , and Her2 3 + or Her2 2 + and ISH +)	77	3.1
Triple negative (ER−, PR −, Her2 −)	180	7.3
Grading		
G1	289	11.7
G2	1666	67.2
G3	514	20.7
Missing	9	0.4
Tumor histology		
NST (no special type)	1870	75.5
Invasive lobular	462	18.6
Other	146	5.9
Clinical stage N		
cN0	1936	78.1
cN1	403	16.3
cN2	102	4.1
cN3	37	1.5

**Table 3:** Multiple logistic regression to assess the association of correct measurement of tumor stage (accordance) with tumor size cT, cN, BMI, age, tumor subtype, grading, histology, Ki67

	Odds ratio	95% Confidence interval	*p*
Clinical tumor stage cT	1.23	(1.10–1.38)	≤ 0.001
Clinical tumor stage cN (cN0 vs. cN +)	1.02	(0.81–1.27)	0.88
BMI	0.997	(0.977–1.02)	0.78
Age	1.00	(0.997–1.01)	0.26
Tumor subtype			0.007
Luminal A (served as reference category)			
Luminal B	0.71	(0.59–0.87)	≤ 0.001
HER2 positive	0.95	(0.54–1.68)	0.86
Triple negative	1.01	(0.68–1.50)	0.97
Grading			0.26
G1 (served as reference category)			
G2	1.23	(0.93–1.64)	1.23
G3	1.09	(0.78–1.51)	1.09
Histology			
NST (served as reference category)			
Invasive lobular	0.96	(0.75–1.21)	0.71
Other	1.09	(0.73–1.64)	0.68
Ki67	1.00	(0.995–1.01)	0.82

All variables were tested in univariable binary logistic regression first

A total of 77 patients had HER2 positive tumors. The overall accuracy of sonography and histology in HER2 positive patients was 79.2% (*n* = 61), overestimation was observed in 10.4% (*n* = 8) of all cases, and underestimation occurred in 10.4% (*n* = 8) of all cases with HER2 positive tumors (see Table 4). Binary logistic regression was performed to proof the association of correct measurement of the tumor stage (accordance) with tumor size cT, cN, BMI, age, grading, histology, Ki67 in HER2 positive tumors (Table 5). All variables were tested in univariable binary logistic regression first, as no variable provided statistical significance, no multiple analysis was performed.

**Table 4:** Clinical and pathological tumor stages of HER2 positive patients

Tumor stage	Clinical stage “cT” (sonography)	Pathological stage “pT” (histology)	Accordance	Underdiagnosis	Overdiagnosis
1a	2 (2.6%)	3 (3.9%)	2 (100%)	0	0
1b	8 (10.4%)	7 (9.1%)	5 (62.5%)	2 (25.0%)	1 (12.5%)
1c	24 (31.2%)	24 (31.2%)	17 (70.8%)	6 (25%)	1 (4.2%)
2	41 (53.2%)	40 (51.9%)	35 (85.4%)	0	6 (14.6%)
3	2 (2.6%)	3 (3.9%)	2 (100%)	0	0
total	77 (100%)	77 (100%)			

Numbers are presented as frequencies (= *n*) and percentages (%). Accordance of tumor stage, underdiagnosis and overdiagnosis refers to clinical stage “cT”

**Table 5:** Binary logistic regression was performed to proof the association of correct measurement of the tumor stage (accordance) with tumor size cT, cN, BMI, age, grading, histology, Ki67 in HER2 positive tumors

	Odds ratio	95% Confidence interval	*p*
Clinical tumor stage cT	1.54	(0.81–2.93)	0.19
Clinical tumor stage cN (cN0 vs. cN +)	0.72	(0.22–2.40)	0.59
BMI	1.06	(0.92–1.23)	0.42
Age	0.98	(0.94–1.02)	0.23
Grading			0.95
G1	–	–	–
G2	1.20	(0.39–3.66)	0.75
G3 (served as reference category)			
Histology			0.90
NST (served as reference category)			
Invasive lobular	1.67	(0.19–14.94)	0.65
Other	–	–	–
Ki67	1.00	(0.97—1.04)	0.82

All variables were tested in univariable binary logistic regression first, as no variable provided statistical significance, no multiple analysis was performed

## Discussion

Tumor size has been identified as a predictive value for the prognosis of breast cancer patients and tumor diameter, as well as lymph node status were found to act as separate but additive prognostic factors [20]. As the therapy landscape evolved during the last years, the accurate determination of the clinical tumor stage by image-guided techniques has become increasingly important. Surgical therapy of invasive breast cancer has changed from radical mastectomy with lymphadenectomy to breast conserving therapy and sentinel-node biopsy [21]. Moreover, the use of neoadjuvant chemotherapy has increased [21, 22]. National and international guidelines are constantly improved to optimize treatment efficacy for the patients while minimizing potential over-treatment and associated toxicities [2, 4, 12]. Therapy decisions are mainly dependent on clinical tumor stage.

Our study demonstrated a high overall accordance (76.5%) of sonographic and histologic tumor stage, while underestimation (14.4%) was more likely to occur than overestimation (9.1%). Sonographic accuracy was dependent on tumor size, with increased accuracy for higher tumor stages. In the present study, the highest accuracy was seen in T2 tumors (82.8%). This is in line with the study of Stein et al. [5]. They conducted a retrospective multicentric analysis of 6,543 patients which showed the highest accuracy for determination of T2 tumors compared to T1 and T3 [5]. One possible reason for our findings can be attributed to the tumor size itself. The classification of the T1 stages encompasses only a small range of millimeters (T1a: < 5 mm, T1b: 5 to < 10 mm, T1c: 10 to < 20 mm), whereas T2 tumors span a larger range (20 to < 50 mm) [3]. In contrast to this, Vijayaraghavan et al., observed the highest accuracy between sonography and pathology in pT1 tumors [23]. However, Vijayaraghavan et al. analyzed only lobular carcinomas [23]. Previous studies showed that accuracy of sonography might be decreased for invasive lobular carcinomas compared to invasive ductal carcinomas and that for invasive lobular carcinomas, mammography or MRI provided better accuracy [5, 24].

Moreover, the histologic subtype might influence sonographic features [25–27]. For example, Yang et al. observed that micro-lobulated mass margins were more frequent in triple negative carcinomas compared to other subtypes [25]. In the present study, we saw that accuracy of sonography was improved in luminal A carcinomas compared to luminal B carcinomas. Like our results, the study of Azhdeh et al. showed that luminal A tumors exhibited the highest accuracy between imaging and pathology, although MRI was utilized to determine clinical tumor stage [28]. Regarding the HER2 positive subtype, Ko et al. observed lower sonographic accuracy [29]. This contrasts with our results. Moreover, we could not find any association between sonographic accuracy in HER2 positive patients, and demographic characteristics, or tumor-related factors. To the best of our knowledge, this is the first study evaluating this association, as in the available literature studies focus on the imaging accuracy in HER2 positive patients after neoadjuvant chemotherapy [30, 31].

The limitations of the study are mainly due to the retrospective nature of our study. Sonography is operator dependent. We had no second, independent ultrasound evaluation by another physician, and therefore, interobserver variability cannot be excluded. Moreover, we have no comparison to other imaging techniques. However, sonography was carried out by board-certified breast physicians with high expertise. Therefore, we focused on ultrasound only, as the most widely available tool in the assessment of breast cancer. Furthermore, we did not provide any information about breast density in imaging according to the guidelines of the American College of Radiology (ACR) [19], nor did we assess the impact of varying breast densities on our findings. Also, we only focused on accuracy of clinical and pathological T stages, rather than on specific measurements or differences in absolute tumor size (mm). This approach may obscure potential inaccuracies within a small range of millimeters. However, prioritizing the T stage over precise tumor size is justified, particularly in the context of planning therapeutic approaches.

## Conclusion

In summary, breast ultrasound is a useful and accurate method in preoperative staging. Sonography is rapid to perform, inexpensive, noninvasive and a fast technique. Our study found ultrasound to be a reliable method in clinical tumor staging, with increased accuracy in higher tumor stages and luminal A tumors. Precise evaluation of the maximum tumor diameter has become essential in guiding the appropriate treatment for breast cancer patients, aiming to avoid the need for re-excision following breast surgery or to determine the necessity of neoadjuvant chemotherapy.

## Data Availability

The datasets generated during the current study are available on reasonable request.

## Abbreviations

WHO: World health organization
HR: Hormone receptor
ER: Estrogen receptor
PR: Progesterone receptor
HER2: Human epidermal growth factor 2
NST: No special type
